# miR-182, miR-221 and miR-222 are potential urinary extracellular vesicle biomarkers for canine urothelial carcinoma

**DOI:** 10.1038/s41598-024-69070-7

**Published:** 2024-08-02

**Authors:** Jenni Karttunen, Lajos Kalmar, Andrew Grant, Jun Ying, Sarah E. Stewart, Xiaonan Wang, Fiona Karet Frankl, Tim Williams

**Affiliations:** 1 Department of Veterinary Medicine, University of Cambridge, Cambridge, UK; 2 Department of Equine and Small Animal Medicine, Faculty of Veterinary Medicine, University of Helsinki, Helsinki, Finland; 3 MRC Toxicology Unit, University of Cambridge, Cambridge, UK; 4 Faculty of Medical Laboratory Science, College of Health Science and Technology, Shanghai Jiao Tong University School of Medicine, Shanghai, China; 5 Department of Biochemistry and Chemistry, La Trobe Institute for Molecular Science, La Trobe University, Melbourne, Australia; 6 School of Public Health, Shanghai Jiao Tong University School of Medicine, Shanghai, China; 7 Cambridge Institute for Medical Research, University of Cambridge, Cambridge, UK

**Keywords:** Tumour biomarkers, Urological cancer, Medical research, Oncology, Urology

## Abstract

Current diagnostic methods for canine urothelial carcinoma (UC) are technically challenging or can lack specificity, hence there is a need for novel biomarkers of UC. To this end, we analysed the microRNA (miRNA) cargo of extracellular vesicles (EVs) from urine samples of dogs with UC to identify candidate miRNA biomarkers. Urine was fractionated using ultrafiltration combined with size-exclusion chromatography and small RNA sequencing analysis was performed on both the EV enriched and (EV free) protein fractions. A greater number of candidate miRNA biomarkers were detected in the EV fraction than the protein fraction, and further validation using droplet digital PCR (ddPCR) was performed on the EV enriched fraction of a second cohort of dogs with UC which indicated that miR-182, miR-221 and miR-222 were significantly overrepresented in dogs with UC when compared with healthy dogs and dogs with urinary tract infections. Pathway analysis confirmed that these three miRNAs are involved in cancer. In addition, their potential downstream gene targets were predicted and PIK3R1, a well-known oncogene is likely to be a shared target between miRNA-182 and miRNA-221/222. In summary, this study highlights the potential of urinary EV-associated miRNAs as a source of biomarkers for the diagnosis of canine UC.

## Introduction

The diagnosis of urothelial carcinoma (UC), the most common type of bladder cancer in dogs^1^, is usually made on cytology from the tumour obtained by traumatic catheterisation, however this diagnostic method is invasive and not easily achieved in first-opinion veterinary practice. Hence, the development of non-invasive urine based diagnostic testing would be beneficial to allow early screening and diagnosis of dogs for UC. Several studies have investigated a variety of potential biomarkers and non-invasive diagnostic tests for canine UC, including protein biomarkers^2,3^, metabolomic biomarkers^4,5^ and DNA copy number aberrations^6^. However, the investigated biomarkers have been inadequately sensitive and/or specific to develop as diagnostic tests for UC. The most promising biomarker for UC available at present detects mutations in the BRAF gene (variant V595E) in cells obtained from urine samples of dogs^7^, however the test can have a sensitivity as low as 64%^8^, and hence false negative results will occur. Thus, there remains a need to identify highly sensitive non-invasive biomarkers for canine UC.

In other fields, there is increasing interest in microRNA (miRNA)-based biomarkers, and changes in miRNA expression might also provide insights into the pathophysiological mechanisms underpinning disease processes, such as carcinogenesis. So far, two articles have reported altered miRNA profiles in canine bladder tissue in UC^9,10^: Vinall et al. compared the miRNAome of transitional cell carcinoma with healthy dogs and dogs with inflammatory bladder disease, while Varvil et al. compared the miRNAome of normal canine urothelium to that of invasive UC. Both studies detected a few differentially expressed (DE) miRNAs in canine UC tissue, but the actual miRNAs differed. Subsequent urinary qPCR analysis of five differentially expressed miRNAs from one of the studies^9^ confirmed significantly reduced abundance of miR-103b and miR-16 in urine samples from dogs with UC compared to other lower urinary tract diseases^11^. Hence, miRNAs do appear to be differentially expressed in canine UC, although further studies are needed to establish if these are robust enough for clinical use.

Extracellular vesicles (EVs) are nanoparticles released by all cells, which contain proteins, mRNAs and miRNAs from the cell of origin^12^. EVs carry intercellular messages, especially in cancer, and therefore their cargo has a high biomarker potential compared to the original biofluid^13–15^. The majority of urinary EVs come from the kidney, prostate and bladder^16^, and so urinary EV-associated miRNAs should be specific for changes in the miRNome of the urinary tract. In addition, the lipid bilayer that surrounds the miRNA cargo of EVs will protect against degradation in the urine by RNAses, hence urinary EV-associated miRNAs should also be more sensitive for changes in the urinary miRNAome. However, large quantities of small RNAs co-isolate with non-vesicular urinary proteins^17^, which may or may not be representative of changes in the miRNAome of the urinary tract, therefore the use of techniques that separate EVs from non-vesicular proteins are important to optimise miRNA biomarker discovery. To date only one study has evaluated urinary EVs from dogs with UC, although this study investigated the abundance of Nuclear Matrix Protein-22 in urinary EVs of dogs with UC^18^, hence the potential of urinary EV-associated miRNAs as a source of biomarkers for canine UC is to date unexplored.

The aim of the present study was to examine EV-associated and non-vesicular (protein-associated) miRNAs as potential biomarkers for canine UC.

## Results

The results of the study are reported in accordance with the ARRIVE guidelines.

### Clinical cases

The ‘sequencing’ cohort included 12 dogs with UC, 5 dogs with urinary tract infections (UTI) and 6 healthy control (HC) dogs. The ‘validation’ cohort included 8 dogs with UC, 8 dogs with UTI and 5 HC dogs. Signalment data for the groups are shown in Table 1.

**Table 1:** Baseline data for dogs in each of the cohorts included in the study.

Group	Age (median [range])	Sex	Breed	Total urine creatinine of sample volume from which EVs were extracted (median [range])	Small RNA content of 500uL of EV fraction (median [range])	miRNA content of 500uL of EV fraction (median [range])
Sequencing UC (n = 12)	10 [8–14] years, 1 unknown	1 male entire 5 male neutered 6 female neutered	1 each of Basset de Bretagne, Staffordshire Bull Terrier, Jack Russell Terrier, Schnauzer, Scottish Terrier, Labrador, Polish Lowland Sheepdog, Pug, Border Collie, Poodle, Weimaraner, Cross-breed	17 [4–90] µmol	17 [2–53] ng	10 [1–18] ng
Sequencing UTI (n = 5)	9 [6–10] years, 1 unknown	1 male neutered 1 male unknown status 2 female neutered 1 female entire	2 Cocker Spaniel, 1 each of German Shepherd, Springer Spaniel and Boxer	27 [2–106] µmol	13 [6–80] ng	2 [2–30] ng
Sequencing HC (n = 6)	5 [2–9] years	4 male entire 1 male neutered 1 female neutered	1 each of Finnish Spitz, Bichon Frise, Labrador, Dachshund, German Shepherd and Cross-breed	59 [30–99] µmol	12 [8–33] ng	6 [3–16] ng
Validation UC (n = 8)	12 [11–13] years	4 male neutered 3 female neutered 1 unknown	2 Labradors, 2 Staffordshire Bull Terriers, 2 West Highland White Terriers, 1 each of Papillon and Beagle	26 [13–30] µmol	26 [17–65] ng	12 [8–40] ng
Validation UTI (n = 8)	7 [0.25–10] years	1 male entire 1 male neutered 1 female entire 4 female neutered 1 unknown	2 Labrador, 1 each of Border Collie, Hungarian Viszla, Golden Retriever, Newfoundland, Staffordshire Bull Terrier and Pug	19 [9–54] µmol	24 [7–38] ng	9 [3–21] ng
Validation HC (n = 5)	8 [2–12] years	3 male neutered 2 female neutered	2 Cocker spaniels, 1 each of Boxer, Border Terrier and Pug	70 [26–82] µmol	27 [10–33] ng	11 [< 1–17] ng

### EV characterisation

**Figure 1:**
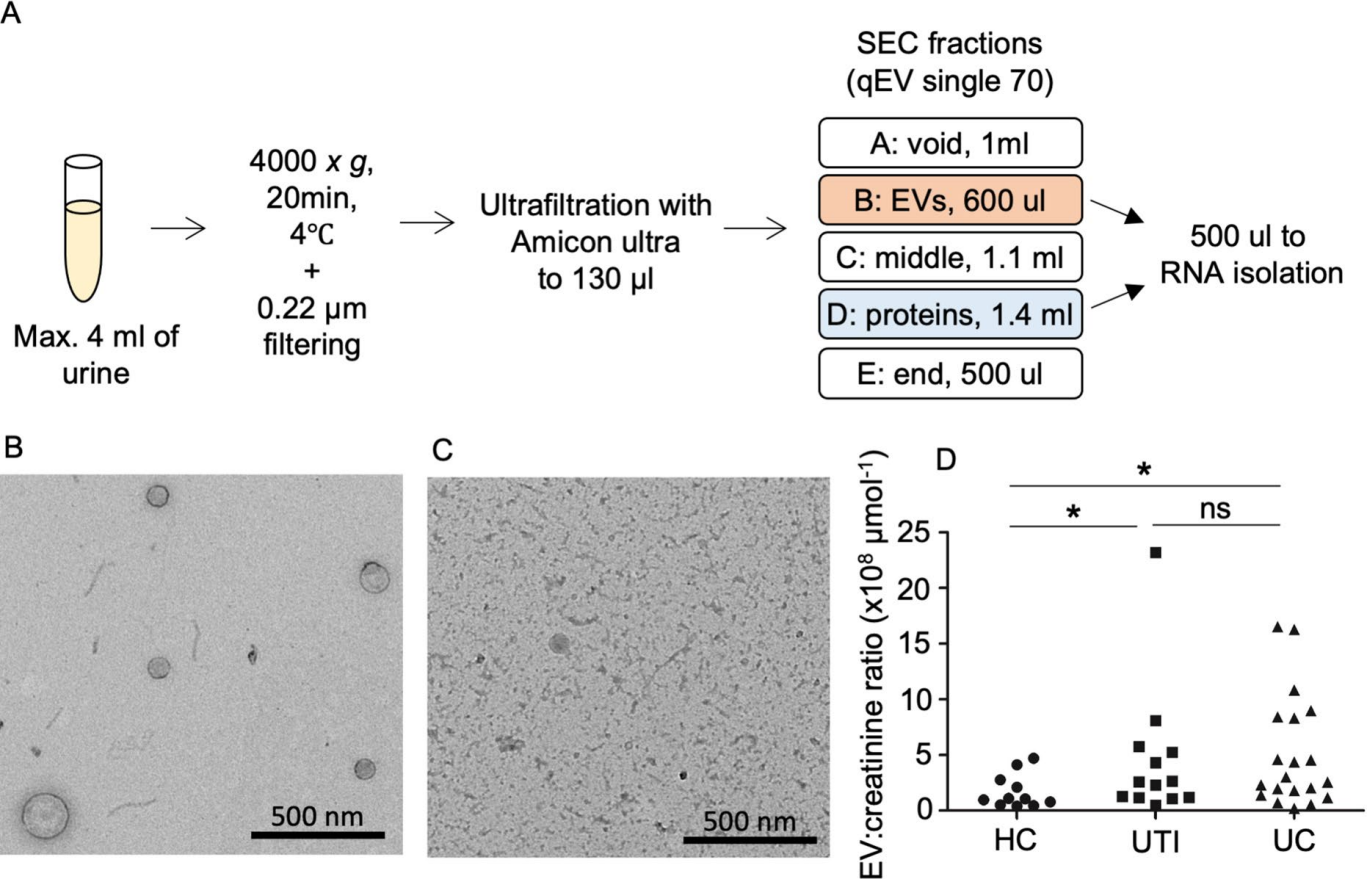
Schematic presentation of the EV isolation steps, and SEC fractions collected during purification. Transmission electron microscopy images of **(B)** Fraction B including EVs and **(C)** Fraction D including vesicle-free proteins. **(D)** Scatter plot of urinary EV numbers normalised to total creatinine content of sample (µmol). Dogs with UTI and UC had significantly higher numbers of urinary EV compared to dogs in the HC group: there was no significant difference between UC and UTI groups. *p < 0.05.

Extracellular vesicles were separated from vesicle free proteins from canine urine as per our previously published protocol using ultrafiltration combined with SEC^17^ which was further optimised for canine urine (Fig. 1A, Supplementary Fig. 1). Transmission electron microscopy imaging (TEM) confirmed the presence of appropriately sized vesicles in fraction B (Fig. 1B) whereas non-vesicular protein fraction D had no visible vesicles (Fig. 1C).

Nanoparticle tracking analysis confirmed the presence of particles with a size profile consistent with EVs (Supplementary Fig. 1C). Urine EV: urine total creatinine ratio (of ‘sequencing’ and ‘validation’ cohorts combined) were significantly greater in dogs with UC (P = 0.04) and UTI (P = 0.03) compared to healthy controls, however there was no significant difference between the UC and UTI groups (P = 0.63) (Fig. 1D).

### Small RNA sequencing revealed differentially expressed miRNAs in UC

For the sequencing cohort samples, RNA from EV and protein SEC-fractions was subjected to small RNA sequencing. First, the size profiles of the two libraries collected for each sample were compared for both length and clustering analysis and the profiles were very similar (Supplementary Fig. 2). The libraries were combined for each sample to maximise read number and the sensitivity for detection of candidate biomarkers in the later analyses. A total of 569,062,706 reads were obtained with a mean of 10,161,834 reads per sample (Supplementary Table 1). Out of processed and filtered 16–24 nt long reads, 1–29% (median 6.3%) aligned to mature canine miRNAs in EV fractions and 0.06–8% (median 0.5%) in protein fractions (Supplementary Table 1). In total, of 455 known mature canine miRNAs in miRbase^19^, 159 were identified from the samples. Of these, 158 were identified in the EV fractions and 150 were present in the protein fractions.

**Figure 2:**
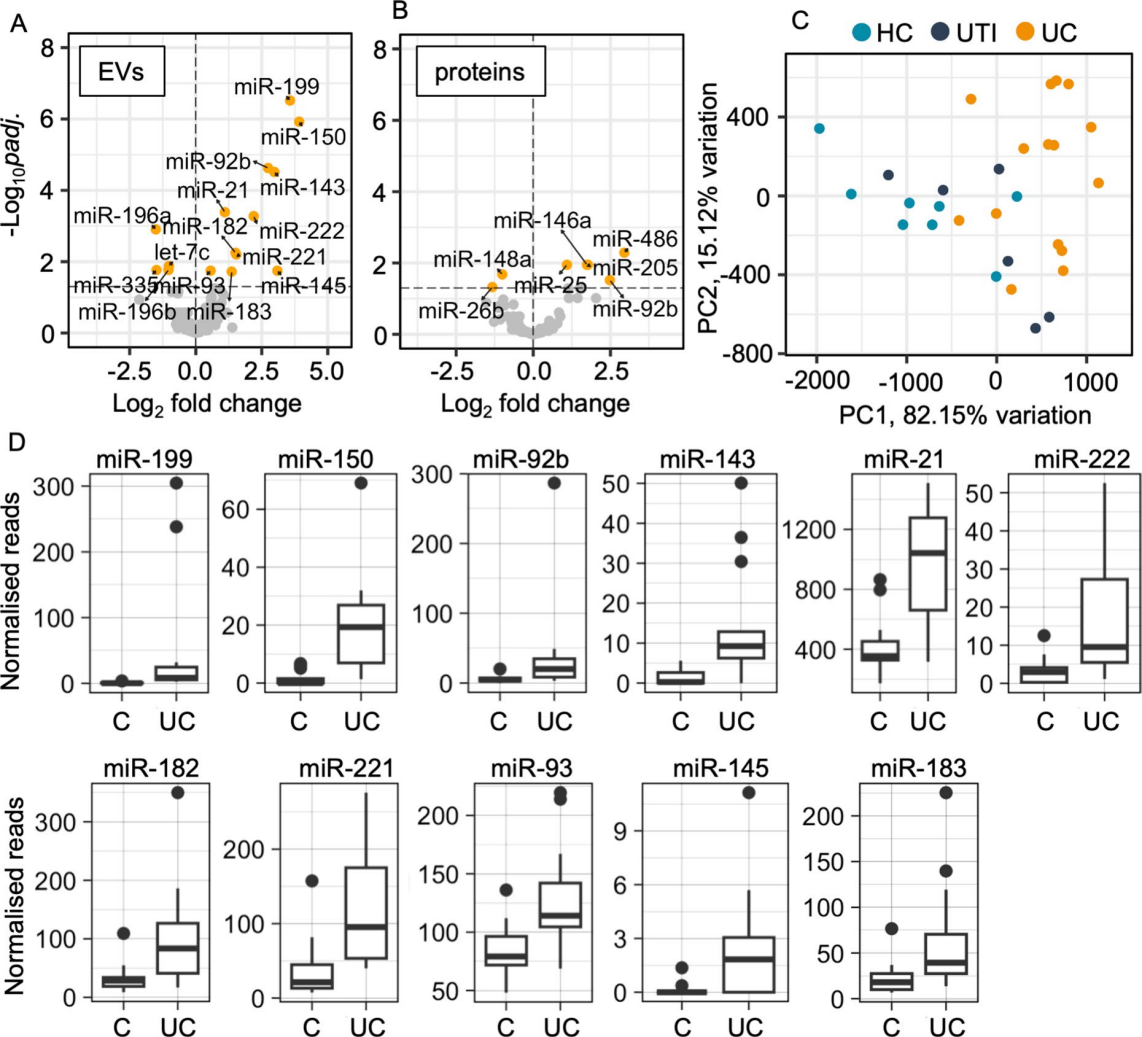
Small RNA sequencing results. Volcano plot of differentially expressed (DE) miRNAs in (**A**) EV fraction and (**B**) protein fraction when UC samples were compared to controls (healthy controls and dogs with UTI). DE-miRNAs with a log_2_ fold change value < 0 were less abundant in UC dogs relative to healthy controls and those with a log_2_ fold change value > 0 were more abundant in UC dogs relative to healthy controls. (**C**) Principal component analysis using all DE miRNAs from EV fraction separated UC samples from HC and UTI groups. (**D**) Normalised read numbers of each DE miRNA in EV fractions.

Differential expression analysis was performed separately for EV and protein fractions from the sequencing cohort. For each analysis, the HC and UTI groups were combined as a control group, and miRNA abundance was compared with that of the UC group. The EV fraction included 11 miRNAs that were more abundant and 4 miRNAs that were less abundant in the UC group (adjusted p-value < 0.05) (Fig. 2A, Supplementary Table 2), hereafter defined as the differentially abundant miRNAs (DA-miRNAs). Protein fractions included 5 significantly upregulated mature miRNAs and 2 downregulated miRNAs in the UC group (Fig. 2B, Supplementary Table 3). All the miRNAs with differential abundance in the protein group had base mean 60 or lower, indicating relatively low expression level, whereas 7 out of 15 DA-miRNAs in the EV fraction had base mean higher than 60. In addition, as the EV fraction yielded a higher percentage of reads aligned to miRNAs and a greater number of DA-miRNAs than the protein fraction, we chose to continue to further analyse the EV fractions only. Principal component analysis (PCA) using the DA-miRNAs from the EV fraction separated the control groups from UC samples (Fig. 2C). Normalised read counts for the DA-miRNAs in the EV fraction are presented in Fig. 2D.

### ddPCR validation of candidate miRNAs

**Figure 3:**
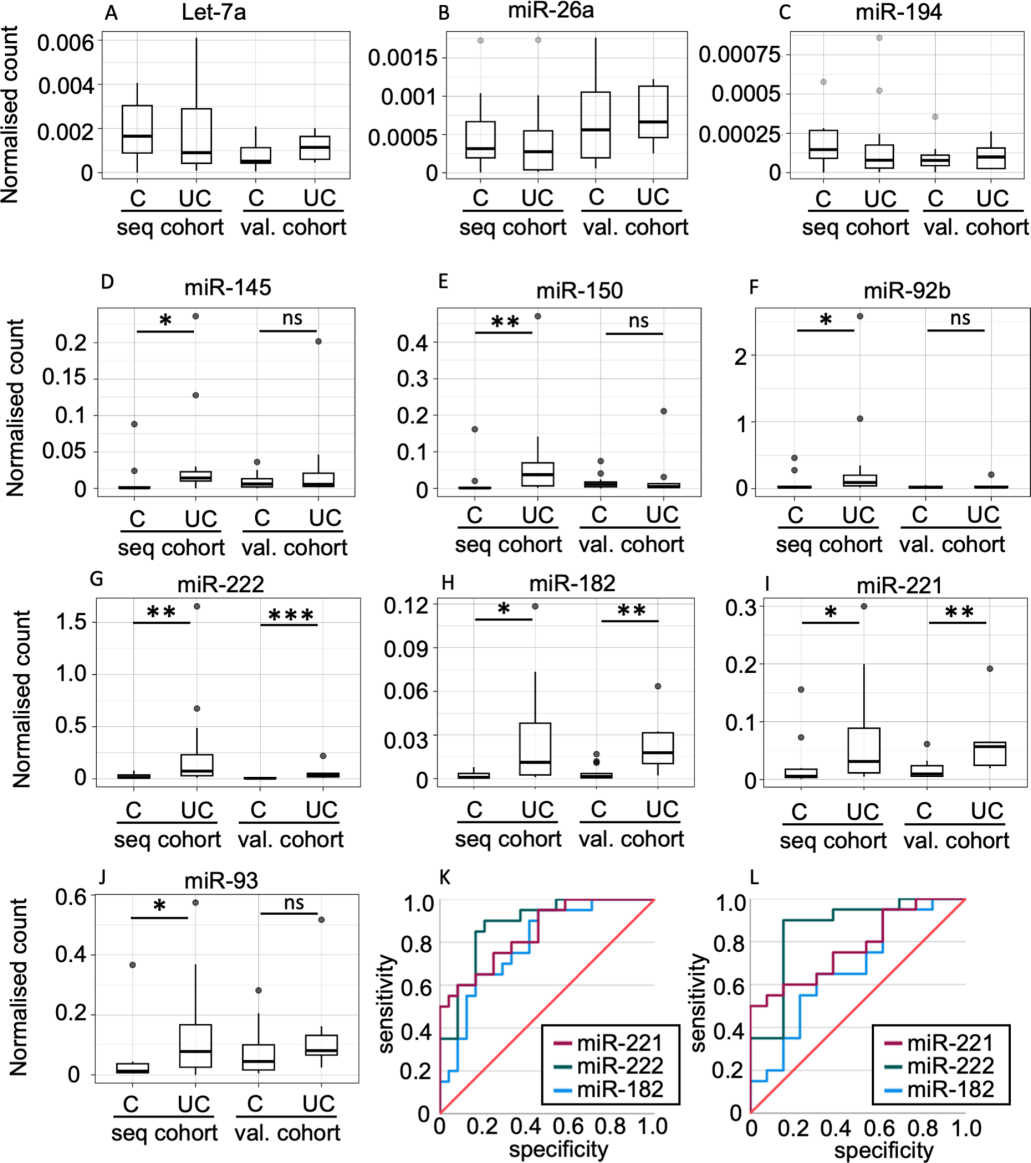
ddPCR validation using both the discovery cohort and validation cohort. (**A**–**C**) Three baseline miRNAs used in normalisation of the potential biomarker miRNAs. Droplet count was normalised to the number of particles in EV fraction/µmol creatinine in the urine. (**D**–**J**) Droplet counts of potential biomarker miRNAs normalised to the closest baseline miRNA and total µmol of creatinine. Receiver operating characteristic curves of the three validated miRNAs with (**K**) UC vs. healthy controls and UTI combined and (**L**) UC vs. UTI samples. *p < 0.05, **p < 0.01, ***p < 0.001.

In addition to potential miRNA candidate biomarkers, the sequencing data from the EV fractions were explored to identify miRNAs that had similar expression across the UC and healthy control groups, and from this group we selected individual miRNAs with high, medium or low expression level against which to normalise our candidates appropriately. As a result, three miRNAs with different expression levels were selected: cfa-let-7a (mean expression: 8702 reads, adjusted *p*-value: 0.55), cfa-miR-26a (mean expression 609 reads, adjusted *p*-value: 0.28) and cfa-miR194 (mean expression 40 reads, adjusted *p*-value: 0.51). Their abundance was further explored with ddPCR which confirmed stability of expression of these miRNAs across the cohorts (Fig. 3A–C). We performed ddPCR for these normalisation miRNAs, in both the sequencing and validation cohorts.

Validation with ddPCR was performed for nine candidate biomarker miRNAs in the EV fraction: miR-92b, miR-93, miR-143, miR-145, miR-150, miR-182, miR-199, miR-221 and miR-222. Of these, miR-199 and miR-143 had low droplet count in ddPCR, meaning no or few individual droplets per sample were detected, therefore, we excluded those miRNAs from further analysis. However, for the other candidate miRNAs the ddPCR data were normalised to the normalisation miRNA with the closest expression level to that of the candidate miRNA and a second normalisation to the total creatinine amount in the sample was then performed (Fig. 3D–J). In the sequencing cohort, the ddPCR results confirmed significant upregulation of the miRNAs of interest (*p* < 0.05).

In the independent validation cohort, three miRNAs (miR-182, miR-221 and miR-222) demonstrated increased expression in the urinary EVs of UC dogs compared to the control groups (UTI and HC combined, Fig. 3G–I).

The biomarker potential of these three candidate miRNAs was evaluated using receiver operating characteristic (ROC) curve analysis. The analysis included two different comparisons: UC dogs versus control (HC and UTI groups combined) (Fig. 3K) and UC dogs versus UTI dogs (Fig. 3L). Normalised miR-222 had the highest area under the curve (AUC) for discriminating UC dogs from all control dogs (AUC = 0.88, 95% CI 0.78–0.98; P < 0.001) and UC dogs from UTI dogs alone (AUC = 0.86, 95% CI 0.72–1.00; P = 0.001) both indicating excellent discrimination. The normalised and indexed expression of miR-182 and miR-222 had acceptable (moderate to good) discrimination (Table 2).

**Table 2:** Results of receiver operating curve analysis evaluating the diagnostic accuracy of the 3 candidate urinary EV-associated miRNAs as a diagnostic biomarker for urothelial carcinoma (UC).

miR candidate	AUC for UC vs. all controls	95% CI for UC vs. all controls	Sig	AUC for UC vs. UTI	95% CI for UC vs. UTI	Sig
miR-222	0.88	0.78–0.98	*p* < 0.001	0.86	0.72–1.00	*p* = 0.001
miR-221	0.79	0.66–0.92	*p* = 0.001	0.68	0.49–0.87	*p* = 0.09
miR-182	0.85	0.73–0.96	*p* < 0.001	0.78	0.62–0.83	*p* = 0.008

Two control groups were used; all controls represented a population of healthy controls and dogs with diagnosed urinary tract infection (UTI), and UTI represented the group of dogs with UTIs only.

### Predicted gene targets of miR-182, miR-221 and miR-222

**Figure 4:**
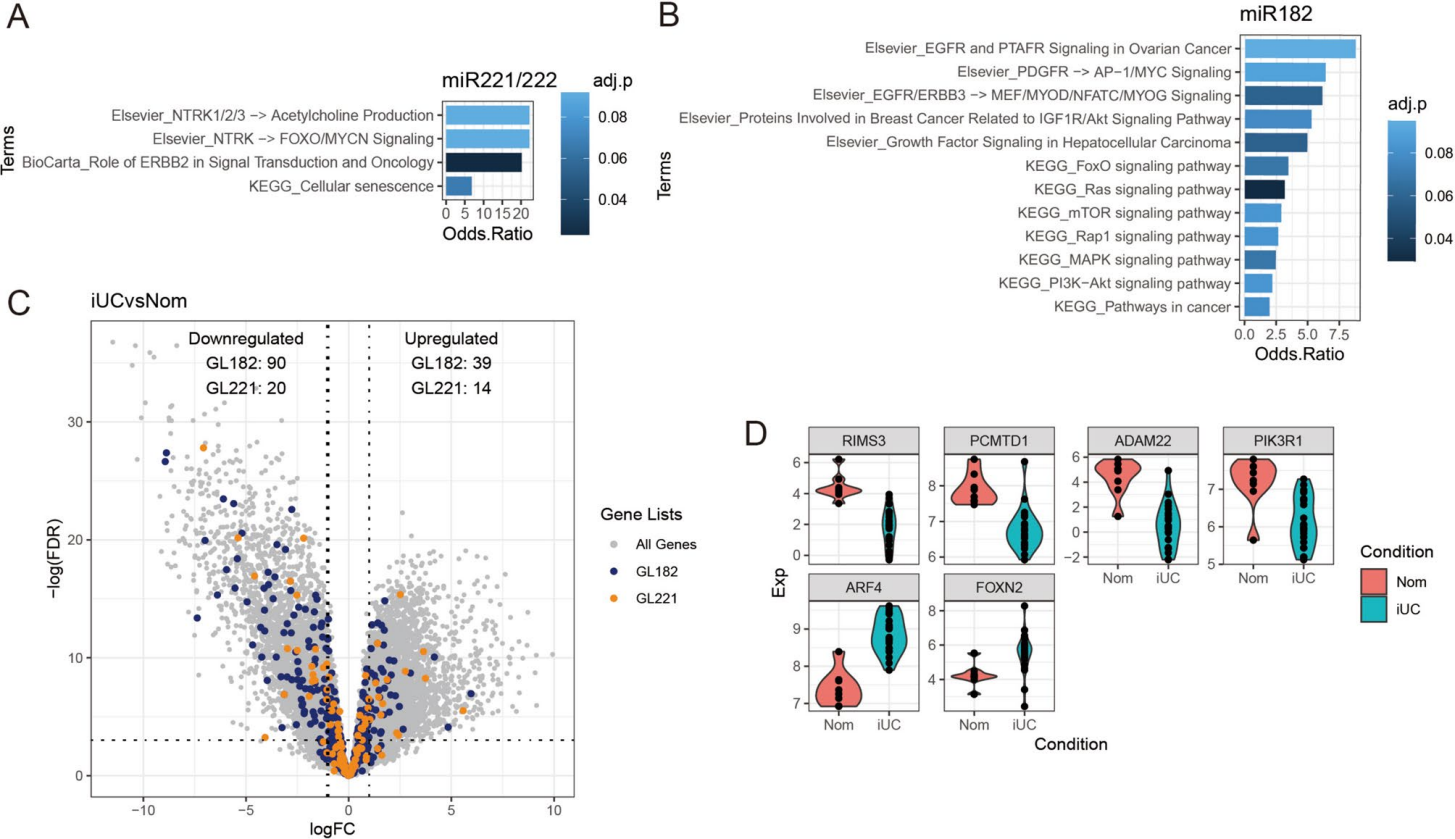
Gene set enrichment analysis of the target genes of miR-182 **(A)** and miR-221/222 **(B)**, respectively, using the Enrichr web tool. Enriched results associated with the signaling pathways from the Elsevier Pathway Collection, the KEGG 2021 Human and the BioCarta 2016 databases were shown. *P* values were adjusted using the Benjamini-Hochberg (BH) method. **(C)** A volcano plot that indicates the differentially expressed (DE) genes between the 22 canine urothelial carcinoma tissue (iUC) samples and the 8 normal control samples (Nom) using the published datasets DRA005844 and SRP217874 obtained from GEO. The overlaps between the DE genes and the miR-182 target genes (GL82) were coloured in blue whereas the ones between DE genes and the miR-221/222 target genes (GL221) were coloured in orange. DE genes were determined using the edgeR Bioconductor package in R. **(D)** Violin plots showing the expression of shared overlapped DE genes between GL182 and GL221/222, which are differentially expressed between the Nom and the iUC samples.

We used both miRDB^20^ and TargetScan v8.0^21^ for biological target prediction for miR-182 and miR221/222 and the overlapped genes were extracted for further analysis, of which 377 genes were found for miR-182 and 121 were found for miR-221/222. Hypergeometric tests were performed to determine to what extent the two databases agreed with each other and the resulting p-values were both less than 0.001, indicating that the number of overlapping genes is more significant than expected. Pathway enrichment analysis using Enrichr^22^ revealed that the target genes of both miR-182 and miR221/222 are highly associated with a wide range of cancer types (Supplementary Fig. 3B and C), including endometrial cancer, ovarian cancer, pancreatic ductal cancer, breast cancer, prostate cancer, glioblastoma and hepatocellular carcinoma with the underlying molecular processes involving the FoxO and the ErbB pathways (Fig. 4A,B).

Since target prediction alone cannot reveal the exact downstream effect of miRNA changes, we collected differentially expressed gene lists from three recently published studies of dog UC^23–25^ and used them to prepare 5 gene sets to further validate our results. The sizes of these gene sets were 30, 342, 361, 888 and 1182 after unrecognised gene symbols were filtered out. The gene targets of miRNA-182 were significantly enriched with down-regulated genes compared between UC and normal (adj.p < 0.05), suggesting that miR-182 is likely to play an inhibitory role in UC. As a comparison, the gene targets of miR-221/222 consist of both up- and down-regulated genes in UC, reflecting a more mixed (activation/inhibitory) role of miRNA-221/222 (Supplementary Fig. 3D).

We also downloaded two available datasets from NCBI SRA (https://www.ncbi.nlm.nih.gov/sra), including 22 bladder tissue samples from dogs with UC and 8 normal controls (Supplementary Fig. 3E). 3451 upregulated and 3269 downregulated genes in UC compared to normal controls were defined with cutoffs of |logFC|< 1 and False Discovery Rate (FDR) < 0.05. Further overlapping with target genes of miRNA-182 and miRNA-221/222 resulted in 129 differentially expressed (DE) genes for gene list 182 (GL182; 39 up-regulated and 90 down-regulated) and 34 DE genes for gene list 221/222 (GL221/222; 14 up-regulated and 20 down-regulated), respectively (Fig. 4C). However, there are only 2 up-regulated (ARF4 and FOXN2) and 4 down-regulated (RIMS3, PCMTD1, ADAM22 and PIK3R1) genes in UC shared between miRNA-182 and miRNA-221/222 (Fig. 4D). Consistent with the pathway enrichment analysis, PIK3R1, a component in the PI3K signalling pathway that is associated with the regulation of cell growth, proliferation and survival^26^, appeared as an important miRNA target gene involved in canine UC development.

## Discussion

The primary aim of the present study was to identify novel urinary EV-based miRNA biomarkers for canine UC, and we identified three candidate miRNA biomarkers, namely miR-182, miR-221 and miR-222. ROC analysis showed that increased normalised abundance of miR-222 was significantly associated with UC and demonstrated excellent discrimination between dogs with UC vs. dogs with UTI and vs. a combined group of healthy dogs and dogs with UTI. Normalised expression of miR-221 and miR-182 associated with EVs was also associated with good discrimination of UC dogs from the other groups.

Detection of the BRAF mutation has shown promise in diagnosis of UC and prostate cancer in dogs^7^ although sensitivity can be as low as 64%^8^, and it is possible that miRNA biomarkers, especially from EVs, could represent complementary detection methods for UC. Future studies comparing the sensitivity and specificity of our individual or combined candidate EV-associated miRNA biomarkers and the detection of BRAF mutations for the diagnosis of canine UC would be of interest.

miR-182, miR-221 and miR-222 are expressed in several tissue types and are not specific to bladder tissue^27^, however previous studies have found a strong correlation between miR-221/222 and cancer^28^ and combination treatment including antagomiRs targeting miR-221-3p and miR-222-3p (along with palladium allyl complex 4d) induced apoptosis of colon cancer and glioblastoma cancer cells^29^, further supporting their potential role in the survival of cancer cells. Another recent study showed that miR-221/222 targeted ANXA protein, regulated cell cycle progression and affected chemosensitivity in breast cancer^30^, which also indicates their importance in tumour pathophysiology. miR-182 is also upregulated in prostate cancer tissues and cells, and lncRNA LINC01018 (which targets miR-182) was shown to regulate its expression^31^. These studies support the biological relevance of increased expression of miR-182, miR-221 and miR-222 within urinary EVs and we hypothesise that the altered abundance of these miRNAs within canine urinary EVs of dogs with UC reflects increased expression of these miRNAs within canine UC cells themselves.

Our target prediction and pathway enrichment analysis revealed that the targets of our candidate miRNA biomarkers are highly associated with cancer tissues, and that the gene targets of our candidate miRNAs are significantly enriched within canine UC gene datasets. Pathway enrichment analysis implicated the FoxO and the ErbB pathways, which regulate cell proliferation, migration, differentiation, apoptosis and cell motility by mediating the PI3K-Akt pathway, the MAPK pathway and the mTOR pathway^32–34^, in canine UC. These data support reports that ErbB/MAPK signalling and the AKT-mTOR-S6K cascade could be therapeutic targets in canine UC^23,35^.

From the key genes overlapping with these cancer-associated pathways, two are shared between miR-182 and miR221/222: *PIK3R1* and *PPP3R1*. Both are well-known oncogenes^36,37^, and according to our prediction results, they could also play an important role in canine UC biology. The findings of our target prediction and pathway enrichment analyses further supports the biological relevance of increased miR-182, miR-221 and miR-222 abundance in urine EVs of canine UC patients and suggests that increased expression of these biomarkers is also apparent within canine UC tissues. Interestingly, the DA-miRNA biomarkers we identified have not been reported in previous studies of the miRNAome of canine UC tissues^9,10^, although similar findings discrepancies have been reported in humans^38^. Our findings are strengthened by the inclusion of a second validation cohort of dogs, which led to the exclusion of several candidate biomarker miRNAs. We hypothesise that the altered abundance of EV-associated miRNAs observed are reflective of changes in the UC cells themselves rather than elsewhere in the urinary tract and further investigation of the expression levels of these candidate miRNAs in canine UC tissues would be of interest.

Unfortunately, two of the miRNAs selected for the ddPCR validation, miR-143 and miR-199, did not produce reliable data due to sensitivity issues with the ddPCR assays available, therefore we were not able to validate their expression by ddPCR or evaluate their potential as a biomarker. We suggest that they are not discounted but require further technical developments, given that both have previously been linked to bladder cancer in humans^39–42^.

One previous study identified reduced abundance of miR-16 and miR-103b in the urine of dogs with UC^11^, however neither of these miRs were identified having reduced abundance in the EV or protein fractions of our UC cohort, which may reflect differences in the sample type evaluated between the two studies (individual EV/protein fractions vs. total urine). Furthermore, in our discovery cohort several miRs were identified as possible biomarkers for UC but most were not differentially abundant in the second independent validation cohort, and so it is possible that miR-16 and miR-103b might not be differentially expressed in other cohorts of dogs with UC.

Currently there is no gold standard method to normalise biomarker or miRNA data from urine or urine EVs^16^, although there is ongoing effort to map out the effects of background variables and normalisation for urine EV studies^43^. Here, we chose to normalise the miRNA abundance determined by ddPCR to three normalisation miRNAs to account for differences in RNA isolation efficiency between samples and we further normalised for urine total creatinine content to adjust for differences in urine concentration and volume between samples. Since the volume and concentration of urine was not standardised among patients, we believe our normalisation strategy should allow for meaningful comparison between the groups whilst also accounting for differences in RNA isolation efficiency.

Since the number of dogs included was small it should be considered a pilot study, and further investigations of the clinical sensitivity and specificity of these miRNA biomarkers in larger cohorts of dogs with UC and UTIs are warranted. In addition, all of the UC dogs we studied presented with clinical signs and had gross evidence of disease on ultrasound, so it would be interesting to investigate if these miRNAs are also useful biomarkers for detecting early stage/microscopic UC, and hence might be useful as a screening test for UC in predisposed breeds (for example West Highland White Terriers).

In conclusion, we identified novel urinary EV-based miRNA biomarkers for canine UC which demonstrated excellent discrimination between dogs with UC vs. either dogs with UTI or a combined group of healthy dogs and dogs with UTI. Further investigation of the clinical sensitivity and specificity of these miRNA biomarkers and the role of these DE miRNAs in the biology of canine UC is warranted.

## Methods

All methods were performed in accordance with the relevant guidelines and regulations. The Ethics and Welfare Committee of the Department of Veterinary Medicine approved the study protocol (CR264).

### Patient recruitment

Dogs with confirmed UC (UC group; based on compatible clinical history, abdominal ultrasound and bladder suction cytology findings) were recruited to the study. In addition, two control groups were included; one was dogs with confirmed urinary tract infections (UTI group) and no clinical suspicion of UC and the second were healthy dogs (owned by staff at our institution) without any clinical history of urinary tract disease and which were not on any medications other than routine parasiticides (HC group). Samples were collected by catheterisation or free catch for the UC group, by cystocentesis for the UTI group and by free catch for the HC group.

Dogs were recruited into two cohorts; the first ‘discovery’ cohort was recruited between July 2017 and November 2019, whilst the second ‘validation’ cohort was recruited between February 2020 and May 2021.

Urine samples were collected and processed within 4 h. Urine was centrifuged at 2000×*g* for 10 min (to pellet cellular components) and the cell-free supernatant harvested prior to freezing at − 80 °C until batch analysis.

### EV isolation and analysis

Frozen urine aliquots were thawed, and a protease inhibitor (Pierce Protease Inhibitor, Thermo Fisher) was added. Extracellular vesicles were isolated from the urine samples by combined ultrafiltration and size-exclusion chromatography (UF-SEC) method, as per our previously published protocol^17^. In brief, urine was centrifuged at 4000×*g*, for 20 min at 4 °C, and the supernatant was filtered using 0.22 µm syringe PES filters (Millex GP, #SLGP033RS). The filtered urine (1–3.6 mL) was concentrated to < 130 µL using Amicon Ultra centrifugal ultrafiltration columns (100 kDa cut off, #UFC810024, Millipore, Burlington, MA, USA). The final volume of concentrated urine was adjusted to 130 µL with PBS and inserted into the size-exclusion chromatography column (qEV single 70 nm, Izon Science Ltd, Burnside, New Zealand) that had been prewashed with 5 mLs of PBS. The SEC fraction volumes were further optimised for dog urine samples which resulted in collection of five fractions: void fraction (Fraction A, 1 mL), EV enriched fraction (Fraction B, 600 µL), second void fraction (Fraction C, 1.1 mL), protein fraction (Fraction D, 1.4 mL) and third void fraction (Fraction E, 500 µL) (Supplementary Fig. 1, Fig. 1A). RNA was isolated from 500 µL of Fraction B and Fraction D; each fraction was combined with 2.5 mL Qiazol lysis reagent (#79306, Qiagen, Venlo, The Netherlands), incubated at room temperature for 5 min and stored at − 70 °C. Remaining SEC fractions were stored at − 70 °C for further EV characterisation.

EV characterisation was performed on Fractions B and D from individual HC dogs. Firstly, transmission electron microscopy (TEM) was performed as previously described^17^ and nanoparticle tracking analysis (NTA) using NanoSight NS300 (Malvern, Worcestershire, UK) as previously described^17^ was also performed on Fraction B of all samples to establish EV concentrations. Particle concentrations (assumed to be EVs) were normalized to urine creatinine concentrations (determined using the pyrogallol red method) as an estimate of 24-h urine EV excretion rate, since measurement of urine protein:urine creatinine concentrations on spot urine samples are highly correlated with 24-h protein excretion in dogs^44^.

### RNA isolation and sequencing library preparation

RNA was isolated from 500 µL of Fractions B and D of the sequencing cohort, and fraction B of the validation cohort samples. Qiazol-sample mixes were defrosted on ice, combined with 0.2 volume of chloroform, and left for 5 min at room temperature. Solutions were centrifuged (12,000×*g*, 15 min, 5 °C), the aqueous phase was collected and combined with 2.5 volumes of 96% ethanol. The sample mix was added to miRNeasy Mini Kit columns (Qiagen, Hilden, Germany, #217004) and washed according to the manufacturer’s instructions. Finally, the total RNA was eluted in 30 μL of nuclease free water. The small RNA profile of isolated RNAs was measured from 1 μL of sample using the Agilent Bioanalyzer Small RNA kit (#5067-1548, Agilent Technologies, Santa Clara, CA, USA). The concentration of 10–40 nucleotide (nt) and 40–80 nt small RNA were calculated using the 2100 Expert software with custom ranges.

Sequencing libraries were prepared from Fractions B and D of the sequencing cohort using QiaSeq miRNA library kit (#331505, Qiagen) combined with QIAseq miRNA 48 Index IL kit (#331595, Qiagen) according to the suggested manufacturer protocol. 99–9600 pg of miRNA sized RNA was used as starting material. Eleven samples were processed and analysed as replicate samples. As an additional step, instead of discarding the magnetic beads after the indexing step as instructed, the beads were collected and washed. As a result, two sequencing libraries from each sample with the same index were obtained: “miRNA library” with the original protocol and “longRNA library”, which included the DNA from the discarded beads. The library preparation failed for one healthy control fraction d sample, and it was excluded from the sequencing run. The samples from each library were combined separately and run in a NovaSeq SP100 flowcell in separate lan.

### Bioinformatics analyses

Bioinformatic analyses were performed with R version 4.2.1. The raw reads including the middle linker sequence were filtered from fastq files using the command line, and the number of unique UMIs for sequences longer than 15 nt in each sample were calculated using a self-written R-script. The results from the two obtained libraries were compared with PCA and correlation analyses (Supplementary Fig. 2) and as a result, the count numbers of both libraries (“mirna” and “long”) were combined for individual samples.

For the analysis of mature miRNAs, the data was further filtered to contain only 16–24 nt read sequences matching mature miRNAs. The alignment of the reads and DE analysis were performed using scripts from DEUS 1.0 pipeline^45^. At first, remaining 16–24 nt long sequences were aligned to mature canine miRNAs from miRbase release 22.1^19^ using blast-based alignment allowing one mismatch and the count numbers for sequences aligned to the same miRNA were combined. Not-aligned sequences were discarded. Differential expression analysis was performed separately for EV and protein fractions, combining HC and UTI groups as a control group, and comparing with the UC samples. During the analysis based on DEseq2 (Version 1.36.0)^46^, replicate samples (libraries prepared from the same RNA) were collapsed together.

### ddPCR validation

Based on the sequencing data, 9 differentially abundant (DA)-miRNAs and 3 stable controls were validated using ddPCR performed using TaqMan miRNA assays described in Supplementary Table 2. Three microliters of 1:3 diluted RNA were converted to cDNA using TaqMan™ MicroRNA Reverse Transcription Kit (#4366596, Thermo Fisher Scientific) according to the manufacturers’ instructions. 1.5 µL of the cDNA were combined with 11 µL of 2X ddPCR Supermix for Probes (#1863024, BIO-RAD), 8.4 µL of nuclease free water and 1.1 µL of assay primer (Supplementary Table 4). Droplets were generated and analysed using the QX200 Droplet Digital PCR System (BIO-RAD).

### Target prediction

The biological targets of candidate miRNAs (miR-182 and miR-221/222) were predicted using two databases, miRDB v6.0^20^ and TargetScan v8.0^21^, since they have predictions specifically for dogs. For miRDB, genes with target scores lower than 60 were filtered out as the default setting, while all of the results from TargetScan were kept. Overlapped genes from the two databases were kept for further analysis, of which 377 genes were found for miR-182 and 121 were found for miR-221/222. A web-based tool Enrichr^22^ was used to analyze these two gene sets for the enrichment of common annotated biological features. Due to limited pathway information specific to dogs, we used general databases for mammals/humans instead, including the Elsevier Pathway Collection, the KEGG 2021 Human^47^ and the BioCarta 2016 in Enrichr.

To confirm if these miRNAs are involved in the regulation of UC progression, we collected DE gene lists from three recently published articles comparing canine UC and healthy controls^23,24,48^ and performed hypergeometric tests to assess the enrichment of these gene lists using the phyper function (Package stats version 4.3.1) in R with the parameter for total target genes (n) given as the mean of the number (18,092.5) of unique target genes in miRDB (16,710) and TargetScan v8.0 (19,475). The final p values were adjusted using the Benjamini-Hochberg (BH) method.

Furthermore, we downloaded the available invasive UC bulk RNA-Seq datasets from NCBI SRA with accession numbers DRA005844^24^ and SRP217874^49^. Differential expression analysis was performed using the edgeR package in R (version 3.42.4). Batch effects between studies were considered in the linear model. Differentially expressed genes were defined as |logFC|> 1 and FDR < 0.05 (adjusted by the Benjamini–Hochberg method). PCA was calculated using the prcomp function in R. Plots were produced using the ggplot2 package (version 3.4.2) in R.

### Statistical analysis

Numerical data were compared between groups using the Mann Whitney U test. For comparison of ddPCR data between groups, the abundance of each candidate miRNA was normalised to the abundance of a selected normalisation miRNA that had a similar expression level to the candidate miRNA. This normalised candidate miRNA abundance was then subsequently normalized to the sample creatinine content, to correct for variation in sample volume used and sample concentration between individuals.

The accuracy of each normalised candidate miRNA (indexed to urinary creatinine content) for distinguishing dogs with UC from dogs with UTI (combining data from ‘sequencing’ and ‘validation’ cohorts) was evaluated using receiver operator curve analysis.

### Supplementary Information

Supplementary Information 1.

Supplementary Information 2.

Supplementary Information 3.

Supplementary Information 4.

## Data Availability

All small RNA sequencing raw data were saved to the NCBI Gene Expression Omnibus (GEO; series accession number GSE256461). To review GEO accession GSE256461: Go to https://www.ncbi.nlm.nih.gov/geo/query/acc.cgi?acc=GSE256461 Enter token mxcpueymftmbfql into the box.

## References

[1] Withrow, S. J., & Vail, D. M. *Withrow & MacEwen's Small Animal Clinical Oncology*. 4th ed. Stephen J. Withrow and David M. Vail. ed. Saunders Elsevier (2007).

[2] Heilmann, R. M. *et al.* Diagnostic performance of the urinary canine calgranulins in dogs with lower urinary or urogenital tract carcinoma. *BMC Vet. Res.* **13**(1), 112 (2017). 28431528 10.1186/s12917-017-1032-5 PMC5401473

[3] Bracha, S. *et al.* A multiplex biomarker approach for the diagnosis of transitional cell carcinoma from canine urine. *Anal. Biochem.* **455**, 41–47 (2014). 24704347 10.1016/j.ab.2014.03.017 PMC4874471

[4] Tsamouri, M. M. *et al.* Untargeted metabolomics identify a panel of urinary biomarkers for the diagnosis of urothelial carcinoma of the bladder, as compared to urolithiasis with or without urinary tract infection in dogs. *Metabolites* **12**(3), 200 (2022). 35323643 10.3390/metabo12030200 PMC8951005

[5] Zhang, J. *et al.* NMR-based metabolomics study of canine bladder cancer. *Biochim. Biophys. Acta* **1822**(11), 1807–1814 (2012). 22967815 10.1016/j.bbadis.2012.08.001

[6] Mochizuki, H., Shapiro, S. G. & Breen, M. Detection of copy number imbalance in canine urothelial carcinoma with droplet digital polymerase chain reaction. *Vet. Pathol.* **53**(4), 764–772 (2016). 26574558 10.1177/0300985815614975

[7] Mochizuki, H., Shapiro, S. G. & Breen, M. Detection of BRAF mutation in urine DNA as a molecular diagnostic for canine urothelial and prostatic carcinoma. *PLoS ONE* **10**(12), e0144170 (2015). 26649430 10.1371/journal.pone.0144170 PMC4674145

[8] Gedon, J. *et al.* BRAF mutation status and its prognostic significance in 79 canine urothelial carcinomas: A retrospective study (2006–2019). *Vet. Comp. Oncol.* **20**(2), 449–457 (2022). 34878687 10.1111/vco.12790

[9] Vinall, R. L., Kent, M. S. & de Vere White, R. W. Expression of microRNAs in urinary bladder samples obtained from dogs with grossly normal bladders, inflammatory bladder disease, or transitional cell carcinoma. *Am. J. Vet. Res.* **73**(10), 1626–1633 (2012). 23013190 10.2460/ajvr.73.10.1626

[10] Varvil, M. S. *et al.* The miRNome of canine invasive urothelial carcinoma. *Front. Vet. Sci.* **9**, 945638 (2022). 36072391 10.3389/fvets.2022.945638 PMC9443663

[11] Kent, M. S. *et al.* MicroRNA profiling of dogs with transitional cell carcinoma of the bladder using blood and urine samples. *BMC Vet. Res.* **13**(1), 339 (2017). 29141625 10.1186/s12917-017-1259-1 PMC5688639

[12] van Balkom, B. W. *et al.* Exosomes and the kidney: Prospects for diagnosis and therapy of renal diseases. *Kidney Int.* **80**(11), 1138–1145 (2011). 21881557 10.1038/ki.2011.292 PMC3412193

[13] Armstrong, D. & Wildman, D. E. Extracellular vesicles and the promise of continuous liquid biopsies. *J. Pathol. Transl. Med.* **52**(1), 1–8 (2018). 29370511 10.4132/jptm.2017.05.21 PMC5784223

[14] Rayamajhi, S. *et al.* Extracellular vesicles as liquid biopsy biomarkers across the cancer journey: From early detection to recurrence. *Clin. Chem.* **70**(1), 206–219 (2024). 38175602 10.1093/clinchem/hvad176 PMC12374260

[15] Mao, Y. *et al.* Role of microRNA carried by small extracellular vesicles in urological tumors. *Front. Cell Dev. Biol.* **11**, 1192937 (2023). 37333986 10.3389/fcell.2023.1192937 PMC10272383

[16] Erdbrügger, U. *et al.* Urinary extracellular vesicles: A position paper by the Urine Task Force of the International Society for Extracellular Vesicles. *J. Extracell Vesicles* **10**(7), e12093 (2021). 34035881 10.1002/jev2.12093 PMC8138533

[17] Karttunen, J. *et al.* Size-exclusion chromatography separation reveals that vesicular and non-vesicular small RNA profiles differ in cell free urine. *Int. J. Mol. Sci.* **22**(9), 4881 (2021). 34063036 10.3390/ijms22094881 PMC8124894

[18] Shin, J. H. *et al.* Changes in urinary exosomal nuclear matrix protein-22 in dogs with urothelial carcinoma: A pilot study. *In Vivo* **38**(1), 190–195 (2024). 38148062 10.21873/invivo.13425 PMC10756486

[19] Kozomara, A., Birgaoanu, M. & Griffiths-Jones, S. miRBase: From microRNA sequences to function. *Nucleic Acids Res.* **47**(D1), D155–D162 (2019). 30423142 10.1093/nar/gky1141 PMC6323917

[20] Chen, Y. & Wang, X. miRDB: An online database for prediction of functional microRNA targets. *Nucleic Acids Res.* **48**(D1), D127–D131 (2020). 31504780 10.1093/nar/gkz757 PMC6943051

[21] Agarwal, V. *et al.* Predicting effective microRNA target sites in mammalian mRNAs. *Elife* **4**, e05005 (2015). 26267216 10.7554/eLife.05005 PMC4532895

[22] Chen, E. Y. *et al.* Enrichr: Interactive and collaborative HTML5 gene list enrichment analysis tool. *BMC Bioinform.* **14**, 128 (2013). 10.1186/1471-2105-14-128 PMC3637064 23586463

[23] Cronise, K. E. *et al.* Characterizing the molecular and immune landscape of canine bladder cancer. *Vet. Comp. Oncol.* **20**(1), 69–81 (2022). 34021685 10.1111/vco.12740 PMC8606617

[24] Maeda, S. *et al.* Comprehensive gene expression analysis of canine invasive urothelial bladder carcinoma by RNA-Seq. *BMC Cancer* **18**(1), 472 (2018). 29699519 10.1186/s12885-018-4409-3 PMC5921755

[25] Elbadawy, M. *et al.* Establishment of an experimental model of normal dog bladder organoid using a three-dimensional culture method. *Biomed. Pharmacother.* **151**, 113105 (2022). 35605292 10.1016/j.biopha.2022.113105

[26] Cantley, L. C. The phosphoinositide 3-kinase pathway. *Science* **296**(5573), 1655–1657 (2002). 12040186 10.1126/science.296.5573.1655

[27] Keller, A. *et al.* miRNATissueAtlas2: An update to the human miRNA tissue atlas. *Nucleic Acids Res.* **50**(D1), D211–D221 (2022). 34570238 10.1093/nar/gkab808 PMC8728130

[28] Di Martino, M. T. *et al.* miR-221/222 as biomarkers and targets for therapeutic intervention on cancer and other diseases: A systematic review. *Mol. Ther. Nucleic Acids* **27**, 1191–1224 (2022). 35282417 10.1016/j.omtn.2022.02.005 PMC8891816

[29] Tupini, C. *et al.* Combined treatment of cancer cells using allyl palladium complexes bearing purine-based NHC ligands and molecules targeting MicroRNAs miR-221–3p and miR-222–3p: synergistic effects on apoptosis. *Pharmaceutics* **15**(5), 1332 (2023). 37242574 10.3390/pharmaceutics15051332 PMC10222778

[30] Kim, J. Y. *et al.* MiR-221 and miR-222 regulate cell cycle progression and affect chemosensitivity in breast cancer by targeting ANXA3. *Exp. Ther. Med.* **25**(3), 127 (2023). 36845963 10.3892/etm.2023.11826 PMC9947582

[31] Luo, W., *et al*. Prognostic value of lncRNA LINC01018 in prostate cancer by regulating miR-182–5p (The role of LINC01018 in prostate cancer). *Nucleosides Nucleotides Nucleic Acids* 1–13. 10.1080/15257770.2023.2298408 (2023). 10.1080/15257770.2023.2298408 38147366

[32] Farhan, M. *et al.* FOXO signaling pathways as therapeutic targets in cancer. *Int. J. Biol. Sci.* **13**(7), 815–827 (2017). 28808415 10.7150/ijbs.20052 PMC5555100

[33] Hynes, N. E. & MacDonald, G. ErbB receptors and signaling pathways in cancer. *Curr. Opin. Cell Biol.* **21**(2), 177–184 (2009). 19208461 10.1016/j.ceb.2008.12.010

[34] Shaw, R. J. & Cantley, L. C. Ras, PI(3)K and mTOR signalling controls tumour cell growth. *Nature* **441**(7092), 424–430 (2006). 16724053 10.1038/nature04869

[35] Riedl, A. *et al.* Comparison of cancer cells in 2D vs 3D culture reveals differences in AKT-mTOR-S6K signaling and drug responses. *J. Cell Sci.* **130**(1), 203–218 (2017). 27663511 10.1242/jcs.188102

[36] Liu, Y. *et al.* Pan-cancer analysis on the role of PIK3R1 and PIK3R2 in human tumors. *Sci. Rep.* **12**(1), 5924 (2022). 35395865 10.1038/s41598-022-09889-0 PMC8993854

[37] Sun, Z. *et al.* Immune-related gene expression signatures in colorectal cancer. *Oncol. Lett.* **22**(1), 543 (2021). 34079596 10.3892/ol.2021.12804 PMC8157333

[38] Fendler, A. *et al.* The translational potential of microRNAs as biofluid markers of urological tumours. *Nat. Rev. Urol.* **13**(12), 734–752 (2016). 27804986 10.1038/nrurol.2016.193

[39] Ye, Q. *et al.* Diagnostic performance of urine and blood microRNAs for bladder cancer: A meta-analysis. *Expert Rev. Anticancer Ther.* **22**(12), 1357–1369 (2022). 36374119 10.1080/14737140.2022.2147511

[40] Asghariazar, V. *et al.* Restoration of miR-143 reduces migration and proliferation of bladder cancer cells by regulating signaling pathways involved in EMT. *Mol. Cell Probes* **61**, 101794 (2022). 35121085 10.1016/j.mcp.2022.101794

[41] Sakaguchi, T. *et al.* Regulation of ITGA3 by the dual-stranded microRNA-199 family as a potential prognostic marker in bladder cancer. *Br. J. Cancer* **116**(8), 1077–1087 (2017). 28324890 10.1038/bjc.2017.43 PMC5396103

[42] Li, X. *et al.* Bladder cancer diagnosis with a four-miRNA panel in serum. *Future Oncol.* **18**(29), 3311–3322 (2022). 36047424 10.2217/fon-2022-0448

[43] Barreiro, K. *et al.* Capturing the kidney transcriptome by urinary extracellular vesicles-from pre-analytical obstacles to biomarker research. *Genes* **14**(7), 1415 (2023). 37510317 10.3390/genes14071415 PMC10379145

[44] Grauer, G. F., Thomas, C. B. & Eicker, S. W. Estimation of quantitative proteinuria in the dog, using the urine protein-to-creatinine ratio from a random, voided sample. *Am. J. Vet. Res.* **46**(10), 2116–2119 (1985). 4062015

[45] Jeske, T. *et al.* DEUS: An R package for accurate small RNA profiling based on differential expression of unique sequences. *Bioinformatics* **35**(22), 4834–4836 (2019). 31228198 10.1093/bioinformatics/btz495 PMC6853685

[46] Love, M. I., Huber, W. & Anders, S. Moderated estimation of fold change and dispersion for RNA-seq data with DESeq2. *Genome Biol.* **15**(12), 550 (2014). 25516281 10.1186/s13059-014-0550-8 PMC4302049

[47] Kanehisa, M. & Goto, S. KEGG: Kyoto encyclopedia of genes and genomes. *Nucleic Acids Res.* **28**(1), 27–30 (2000). 10592173 10.1093/nar/28.1.27 PMC102409

[48] Elbadawy, M. *et al.* Establishment of a novel experimental model for muscle-invasive bladder cancer using a dog bladder cancer organoid culture. *Cancer Sci.* **110**(9), 2806–2821 (2019). 31254429 10.1111/cas.14118 PMC6726682

[49] Parker, H. G. *et al.* RNAseq expression patterns of canine invasive urothelial carcinoma reveal two distinct tumor clusters and shared regions of dysregulation with human bladder tumors. *BMC Cancer* **20**(1), 251 (2020). 32209086 10.1186/s12885-020-06737-0 PMC7092566

