# Supplementary material for: miR-182, miR-221 and miR-222 are potential urinary extracellular vesicle biomarkers for canine urothelial carcinoma
Source: Sci Rep. 2024 Aug 2;14:17967. doi: 10.1038/s41598-024-69070-7 (PMC11297243; doi:10.1038/s41598-024-69070-7)
Supplement: Supplementary file 1 — Supplementary Information 1. [file 41598_2024_69070_MOESM1_ESM.docx]

**Supplementary Figure 1: Optimization of size exclusion chromatography (SEC) fractions.** (A) SEC for dog urine samples was optimised by performing two test sets: the first included 200µL void volume following ten 400µL fractions and the second comprised 600µL void volume and nine 200µL fractions. (B) The optimised SEC fractions used in this study were fractions D-F. (C) Particle concentration of each fraction was measured with nanoparticle tracking analysis (NTA, NanoSight NS300, Malvern, Worcestershire, UK) and size distribution of the particles in the fraction containing the highest number of EVs is shown in the inset. (D) Protein concentration of each fraction measured with BCA assay. (E) Western blot against EV-specific protein TSG-101 (antibody: Abcam ab83) was performed to confirm the presence of vesicles. Western blot was performed as previously described [17].


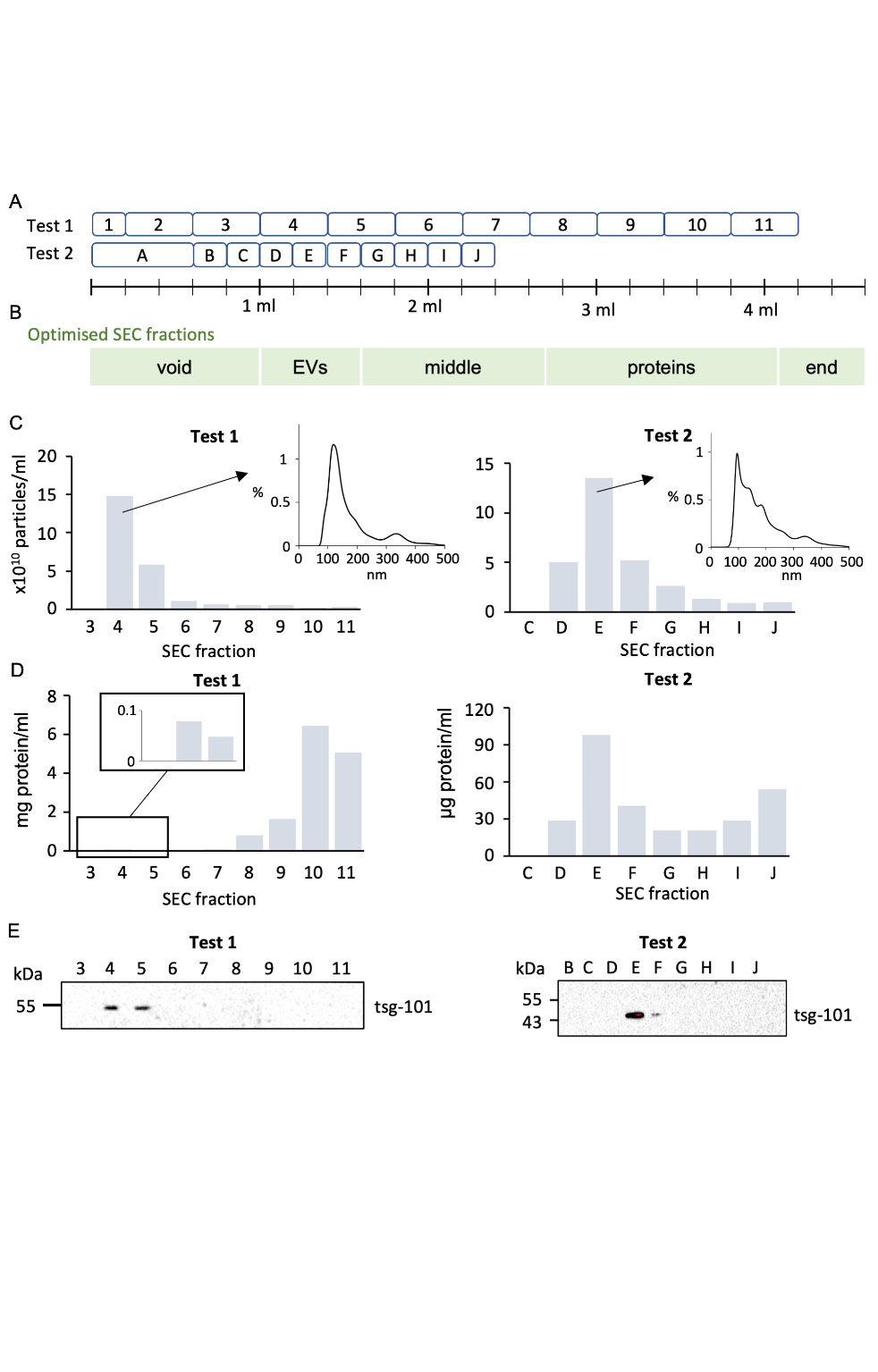


**Supplementary Figure 2: comparison of two sequencing libraries**

Our previous findings from urine EV profiles [17] have shown that EVs include mainly longer ~60 nt RNA molecules and we wanted to test whether it was possible to also collect longer than miRNA-sized RNAs during library preparation. In the library preparation process, size separation is achieved using two magnetic bead separations after the indexes are added: in the first step longer DNAs are bound to the beads and the miRNA-sized library remains in the supernatant. In the second step, the miRNA-sized library is bound to the beads and shorter interfering DNAs remain in the supernatant. Here, we collected and purified the DNA that was meant to be discarded during the first bead purification and hence created two different libraries with the same indexes: “miRNA library” for the correct protocol and “long library” which included the DNA from the first bead purification step. **(A-B)** DNA concentration measured from individual libraries with Qubit X assay. In individual sample levels, the “long library” had higher DNA concentration compared to the “miRNA library”, as expected, however the concentration of DNA obtained using each method did not vary between the sample groups. **(C)** Agilent bioanalyzer High DNA kit measurement from pooled libraries. Both pooled libraries have two peaks in the profile, with the miRNA library sized peak identified at around 180 bp, and the second peak being the main peak in “long library”. The “long library” also had a small shoulder towards the longer lengths and two smaller peaks in the 300 bp area. **(D)** Barplots of the read length distribution after adapter sequences were trimmed out from three individual representative samples. There were different size profiles across the samples, but for individual samples the profile of the “miRNA library” and “long library” were always very similar. **(E)** To further compare the small RNA profiles, a clustering analysis was performed for the normalised trimmed reads. Data are presented for healthy control samples. With minimal outliers, the two libraries from the same sample (marked MIHT*XX* and MIHT*XX*.1) clustered together and had similar small RNA profiles. Based on these comparisons, the reads from the two libraries for individual samples were combined for the actual analysis.


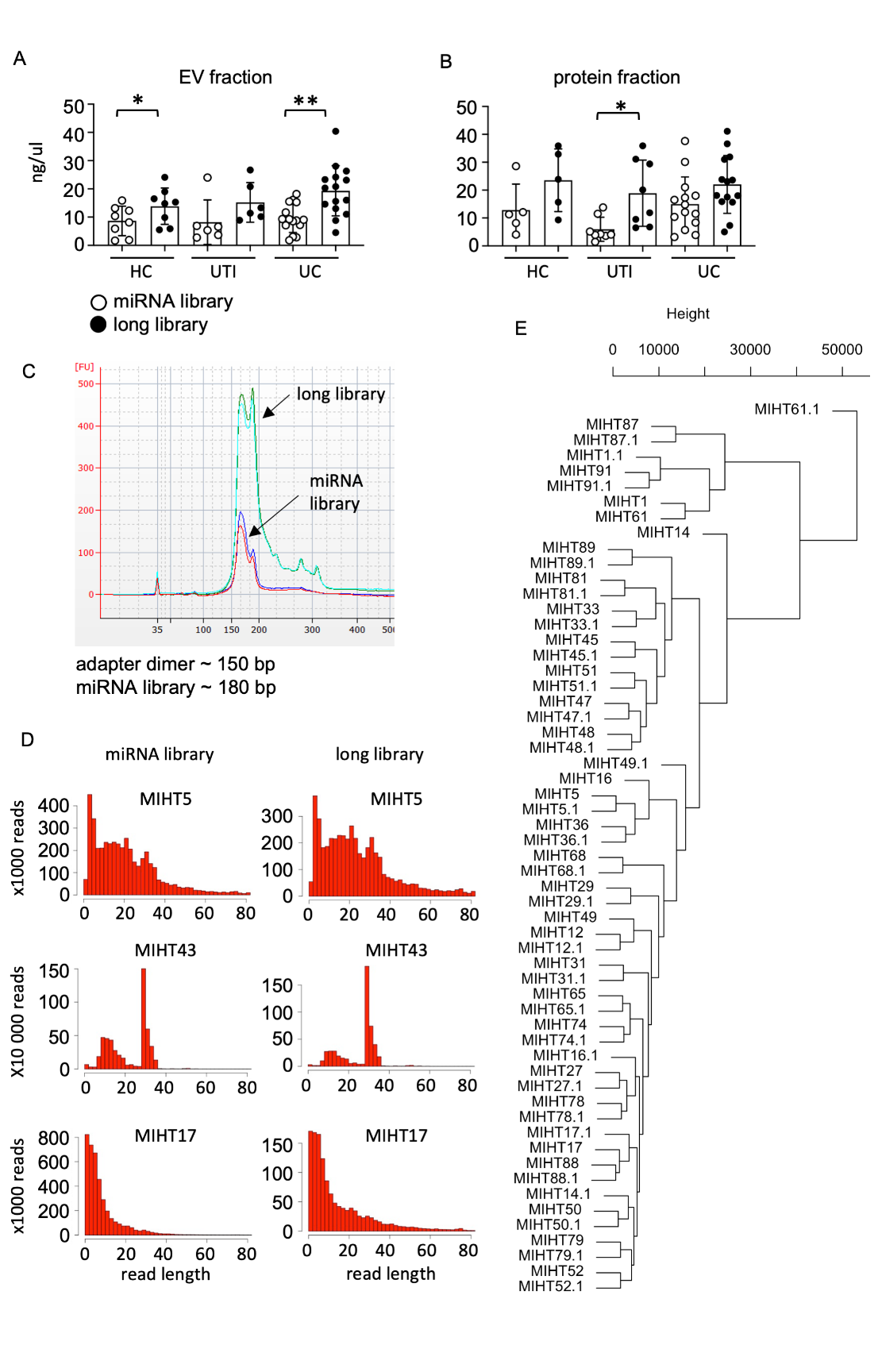


**Supplementary Figure 3.** Gene set enrichment analysis of the target genes of miR-182 **(A)** and miR-221/222 **(B),** respectively, using the Enrichr web tool. Enriched results associated with various types of cancers from the Elsevier Pathway Collection, the KEGG 2021 Human and the BioCarta 2016 databases were shown. P values were adjusted using the Benjamini-Hochberg (BH) method. **(C)** A Venn diagram showing the overlap of the target genes in the enriched cancer-associated pathways of miR-182 and miR-221/222. **(D)** Gene set enrichment analysis was done using the published up- and down-regulated genes between canine UC (iUC) and normal tissues (Nom). The enrichment was assessed using hypergeometric tests and the p values were further corrected using the BH method. **(E)** A PCA plot of the 22 iUC and the 8 Nom samples using the published datasets DRA005844 and SRP217874 obtained from GEO.


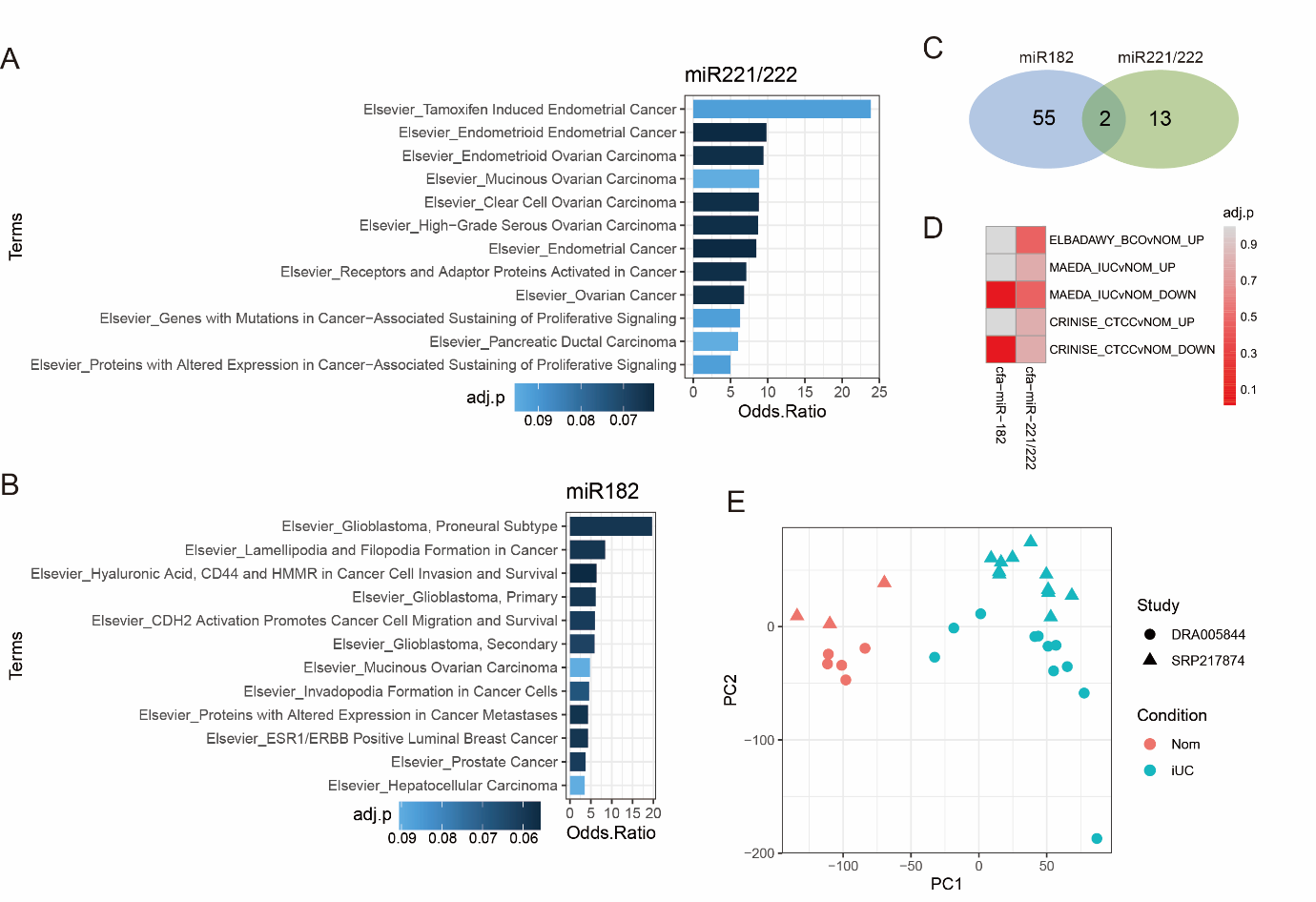


**Supplementary Table 4**: TaqMan primer assays used in the ddPCR validation.

| TaqMan Assay | Target sequence | group |
| --- | --- | --- |
| hsa-miR-26a (#000405) | UUCAAGUAAUCCAGGAUAGGCU | Baseline control |
| hsa-miR-194 (#000493) | UGUAACAGCAACUCCAUGUGGA | Baseline control |
| hsa-let-7a (#000377) | UGAGGUAGUAGGUUGUAUAGUU | Baseline control |
| hsa-miR-150-5p (#00473) | UCUCCCAACCCUUGUACCAGUG | Potential biomarker |
| cfa-miR-199 (#004405_mat) | ACAGUAGUCUGCACAUUGGUU | Potential biomarker |
| hsa-miR-145 (#002278) | GUCCAGUUUUCCCAGGAAUCCCU | Potential biomarker |
| mmu-miR-93 (#001090) | CAAAGUGCUGUUCGUGCAGGUAG | Potential biomarker |
| hsa-miR-143 (#002249) | UGAGAUGAAGCACUGUAGCUC | Potential biomarker |
| hsa-miR-92b (#007028_mat) | UAUUGCACUCGUCCCGGCCUC | Potential biomarker |
| hsa-miR-222 (#002276) | AGCUACAUCUGGCUACUGGGU | Potential biomarker |
| hsa-miR-221 (#001134) | AGCUACAUUGUCUGCUGGGUUU | Potential biomarker |
| hsa-miR-182 (#002334) | UUUGGCAAUGGUAGAACUCACACU | Potential biomarker |
